# Distribution and Determinants of Body Mass Index of Non-smoking Adults in Delhi, India

**Published:** 2007-09

**Authors:** Pragti Chhabra, Sunil K. Chhabra

**Affiliations:** 1 Department of Community Medicine, University College of Medical Sciences, Dilshad Garden, Delhi 110 095, India; 2 Department of Cardiorespiratory Physiology, Vallabhbhai Patel Chest Institute, Delhi 110 007, India

**Keywords:** Body mass index, Obesity, Overweight, Underweight, India

## Abstract

Data on height and weight of 3,428 non-smoking healthy adult subjects, obtained during an earlier community-based study in Delhi, India, on chronic respiratory morbidity due to ambient air pollution was analyzed to study the distribution of body mass index (BMI) and its determinants among adults in Delhi. The sample was drawn by systematic sampling from rural and urban areas of Delhi. In urban areas, the sampling frame was restricted to areas around air quality-monitoring stations. However, the areas were spread across the city and reflected wide economic spectrum. Subjects were classified as underweight, normal, overweight, and obese as per the criteria of the World Health Organization for BMI. The mean BMI of the entire sample was 22.14 ±4.61. It was higher among females, urban residents, and the higher-income group. Overall, 49.7% of the 3,428 subjects had a normal nutritional status, 24.8% were underweight, 19.4% overweight, and 6.1% obese. The prevalence of underweight was higher in rural areas (38.5%) and among the lower-income group (39.9%), while overweight and obesity were more common in urban residents (22.7% and 7.5% respectively), among females (21.7% and 7.7%), and the higher-income group (31.8% and 11%) (p<0.05). The adjusted odds for underweight were 2.02 for rural subjects and 4.00 for the lower-income group. For overweight or obesity, odds were 5.6 for the higher-income group, 3.62 for urban residents, and 2.5 for females. It was concluded that problems of both underweight and overweight and obesity exist among the adults of Delhi. While females, residents of urban areas, and economically-better-off were more likely to be overweight or obese, residents of rural areas and those from lower-income groups were more likely to be underweight.

## INTRODUCTION

Emerging evidence suggests that overweight and obesity are increasing worldwide (1,2). On the other hand, the problem of undernutrition has long been a major public-health concern in developing countries. Recent studies in developing countries have, however, shown that a transition is occurring, and both undernutrition and overweight or obesity could co-exist (3-5).

With sustained economic development, increased availability and consumption of food, changes in life-style, and increased urbanization, India is likely to face similar transitions in nutrition-related problems as other developing economies of the world. While the problem of undernutrition has been well-documented, especially in children but less often appreciated in adults, recent studies have also focused on the problem of overweight and obesity (6,7). As both underweight and overweight increase the risk of several diseases (5,8) and both may co-exist in communities undergoing a transition, knowledge of the magnitude of both the problems becomes an important public-health issue. Although studies on nutritional status have been carried out in India (9-16), the available information has limitations. Some studies either were carried out only in women or were confined to urban or rural areas or limited to specific age-groups. In view of these shortcomings and the emerging scenario of co-existence of underweight and overweight or obesity, further studies on the nutritional profile in the community are required.

Recently, we carried out a community-based study in Delhi to investigate the chronic respiratory morbidity due to ambient air pollution (17). Data on height and weight of non-smoking, healthy subjects from that study were analyzed to describe the nutritional profile of adults in Delhi. We also attempted to quantify the risk associated with economic and demographic factors for both underweight and overweight or obesity.

## MATERIALS AND METHODS

### Sample selection and methodology

The details of sampling have been described earlier (17). The study was carried out during 1996–1998. At that time, we did not have any Institutional Review Board as the practice of a separate ethical approval for research projects did not exist at the time. The Ministry of Environment and Forests, Government of India, approved the project.

Briefly, nine urban areas where air quality-monitoring stations were located were surveyed. Although the sampling frame was, thus, restricted, the areas were spread across the city and reflected a wide economic spectrum, and information on BMI is likely to reflect the actual picture in Delhi. A stratified random sample was taken from each area. Three housing colonies—one each from the lower-, middle- and higher-socioeconomic categories—were selected from each urban area. Similarly, four rural areas were also surveyed. Area maps were drawn, and households were selected by systematic sampling in each of the three colonies. The starting point in an area was randomly chosen, and thereafter at regular intervals, households were included. In each selected house, all the available members aged over 18 years were administered a standardized respiratory symptoms questionnaire. The subjects were measured wearing light clothing and no footwear. Weight was measured to the nearest 0.5 kg using a bathroom scale, which was calibrated on a weekly basis with known weights. To ensure consistency and avoid interobserver variability, a single machine was used, and the same observer took the measurements. Height was measured with the subject standing erect with head in the Frankfurt plane and ankles pressed against a wall on which a measuring tape had been fixed. The study was carried out mostly in the forenoon. Chronic lung diseases and smoking can themselves affect the body mass index (BMI). Therefore, for the present study, we excluded subjects with chronic lung diseases and smoking and included only non-smoking healthy adults identified by history and examination by physicians.

### Definition of categories

BMI was calculated by dividing the weight of an individual in kg by the square of his/her height measured in metres. The subjects were classified into one of the four categories according to the BMI (18): (a) underweight—BMI <18.5 kg/m^2^; (b) normal—BMI 18.5–24.9 kg/m^2^; (c) overweight—BMI 25–29.9 kg/m^2^; (d) obese—BMI ≥30 kg/m^2^. As the 5^th^, 85^th^, and 95^th^ percentiles have also been used for defining underweight, obesity, and overweight subjects, these were also calculated.

Depending upon the monthly family income, the population was classified into three categories: Low: income below US$ 100; Middle: income between $ 100 and 350; High: income above $ 350. Income was assessed by direct questioning.

### Statistical analysis

Data were analyzed using the SPSS software (version 11.0) and GraphPad Prism (version 4.01). The mean BMI ±SD for each category was computed. Student's t-test and ANOVA were used for comparing quantitative data (BMI) between two and three groups respectively. The percentiles were calculated for each five-year age-group. Smoothened curves of percentiles of BMI were plotted against age using the weighting procedure provided in GraphPad Prism (version 4.01). The prevalence of underweight, normal, overweight, and obese subjects was obtained for each category. The chi-square test was applied to study the difference in proportions and to obtain unadjusted odds ratios. Multiple logistic regression analysis was done to calculate the adjusted odds ratio. For income level (3 categories), ordinal logistic regression analysis was used. For the purpose of calculating, the adjusted odds for the occurrence of underweight subjects with BMI <18.5 were compared with those with normal BMI (18.5–24.9), while for overweight and obesity, subjects with BMI ≥25 were compared with those with normal BMI (18.5–24.9).

## RESULTS

**Fig. 1:**
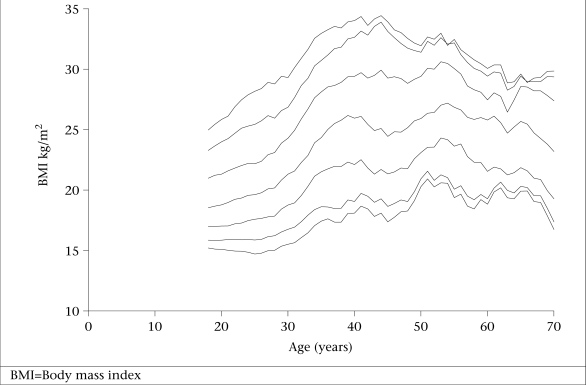
Smoothened curves of 5^th^, 10^th^, 25^th^, 50^th^, 75^th^, 90^th^, and 95^th^ percentiles of BMI plotted against age in males

**Fig. 2:**
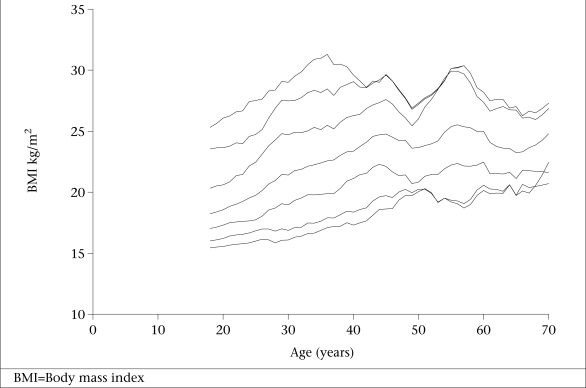
Smoothened curves of 5^th^, 10^th^, 25^th^, 50^th^, 75^th^, 90^th^, and 95^th^ percentiles of BMI plotted against age in females

In total, 3,428 subjects were studied. Table 1 shows the demographics and characteristics of the population. Table 2 depicts the age-wise mean and percentiles of BMI in both sexes. Significant differences (p<0.05) were observed in the mean BMI according to age in both males and females. Subjects in the 18–20-year age-group had the lowest BMI, and those in the 51–55-year age-group had the highest BMI. It decreased in the higher age-groups. Smoothened curves of the 5^th^, 10^th^, 25^th^, 50^th^, 75^th^, 90^th^, and 95^th^ percentiles of BMI plotted against age in males and females are shown in Figures 1 and 2.

**Table 1:** Demographic profile of study subjects

Factor	Males		Females		Total	
	No.	%	No.	%	No.	%
Age (years)						
18–30	751	45.2	912	54.8	1,663	48.6
31–50	425	33.4	847	66.6	1,272	37.1
>50	184	37.3	309	62.7	493	14.3
Sex						
Male	–	–	–	–	1,360	39.7
Female	–	–	–	–	2,068	60.3
Income						
Low	295	34.9	550	65.1	845	24.6
Middle	558	36.5	971	63.5	1,529	44.6
High	507	48.1	547	51.9	1,054	30.7
Residence						
Urban	1,095	41.8	1,525	58.2	2,620	76.4
Rural	265	32.8	543	67.2	808	23.6

**Table 2:** Distribution of percentiles of BMI according to age and sex

Age (years)	Males					Females				
	Mean	5th	50th	85th	95^th^	Mean	5th	50th	85th	95th
18–20	19.06	15.57	18.44	22.04	25.03	19.24	15.05	18.60	22.92	25.25
21–25	19.96	15.87	19.38	23.11	26.51	19.99	15.24	19.56	23.66	27.05
26–30	21.40	16.43	21.13	25.25	27.54	20.86	15.20	20.25	25.14	28.43
31–35	22.14	16.33	21.77	25.92	28.67	22.85	16.36	22.49	27.41	30.83
36–40	23.53	17.21	23.42	26.97	29.89	25.02	17.00	24.74	30.16	33.75
41–45	24.12	17.51	24.28	27.75	30.11	25.20	16.76	24.82	30.49	34.01
46–50	23.85	17.99	23.62	27.10	30.20	24.30	16.08	24.43	29.11	32.50
51–55	26.13	18.17	25.22	32.92	35.76	26.54	18.50	26.34	31.70	34.51
56–60	25.12	16.99	24.86	30.51	32.24	24.25	16.39	24.30	29.68	33.44
>60	24.05	17.53	23.70	27.99	30.82	23.66	16.15	23.73	28.38	31.16

BMI=Body mass indexportions

The mean BMI of the entire sample was 22.14 ±4.61. It was significantly higher in females compared to males (p<0.01), while the BMI of rural residents was significantly lower than that of urban residents (p<0.01). It was lowest in the low-income category and highest in the high-income category (p<0.01) (Table 3). Overall, only half (49.7%) of the 3,428 subjects had a normal nutritional status, while 24.8% were underweight, 19.4% overweight, and 6.1% obese. Table 3 shows the proportion of underweight, normal, overweight, and obese subjects in different categories of sex, income status, and residence. The chi-square test revealed these proportions to be significantly different. While the proportion of underweight subjects were nearly similar between males and females; the overweight and obese subjects were greater among females (p<0.001). Considering residence, the underweight subjects were in a greater proportion in rural areas and, the overweight and obese subjects, in urban areas (p<0.001). For income categories, the underweight subjects were in a significantly greater proportion and the overweight and obese subjects in a lesser proportion in the low-income category, and the reverse was observed in the high-income category (p<0.001).

**Table 3:** BMI and nutritional categories according to sex, residence, and income

Factor	Mean (BMI±SD)	Proportions of subjects			
		Underweight	Normal	Overweight	Obese
Sex					
Male	21.65±4.09	26.0	53.3	16.9	3.8
Female∗	22.46±4.89	24.0	47.4	21.0†	7.7†
Residence					
Rural	20.16±3.70	38.5†	51.1	8.5	1.9
Urban∗	22.75±4.69	20.5	49.3	22.7†	7.5†
Income					
Low	20.16±3.70	39.9†	50.2	8.4	1.5
Middle∗	21.8± 4.50	26.6	51.2	16.8	5.3
High∗	24.15±4.50	10.0	47.2	31.8†	11.0†

∗ p<0.001 (Student's t-test/ANOVA) for comparisons of BMI within categories of sex, residence, and income (compared to low income);

† p<0.001 (chi-square test) for significance of differences in proportions of the four categories of nutritional status according to sex, residence, and income;

ANOVA=Analysis of variance; BMI=Body mass index; SD=Standard deviation

Finally, multiple logistic regression analysis was carried out to quantify the adjusted odds (with 95% confidence interval [CI]) with different determinant variables for underweight and overweight or obesity status (Table 4). For underweight status, area of residence and income were significant determinants. The adjusted odds ratio for rural residence was 2.02 (95% CI 1.67–2.44) compared to urban. The odds ratio for the low-income category was 4.00 (95% CI 3.10–5.16), and for the middle-income category, it was 2.07 (95% CI 1.62–2.65) compared to the high-income category. For overweight or obesity status, income category, residence, sex, and age were significant determinants. The odds ratio for the high-income category was 5.61 (95% CI 3.08–10.19) and for the middle-income category, it was 3.73 (95% CI 2.05–6.79) compared to the low-income category. The adjusted odds ratio for urban residence was 3.62 (95% CI 2.09–6.30) compared to rural residence. The female subjects had odds of 2.52 (95% CI 1.80–3.52) compared to the males subjects.

**Table 4:** Adjusted odds ratios for underweight, overweight, and obesity obtained from logistic regression analysis

Factor	Adjusted odds ratio for underweight	Adjusted odds ratio for overweight and obesity (95% CI)
Age	0.95 (0.94–0.96)∗∗	1.03 (1.02–1.04)∗∗
Sex		
Female	1.07 (0.90–1.30)NS	2.52 (1.80–3.52)∗∗
Male	1	1
Area of residence		
Rural	2.02 (1.67–2.44)∗∗	1
Urban	1	3.62 (2.09–6.30)∗∗
Income level		
Low	4.00 (3.10–5.16)∗∗	1
Middle	2.07 (1.62–2.65)∗∗	3.73 (2.05–6.79)∗∗
High	1	5.61 (3.08–10.19)∗∗

∗∗ p<0.001; 1=Reference category; CI=Confidence interval; NS=Not significant, p>0.05

## DISCUSSION

The present study has shown that both underweight and overweight or obesity co-exist in the adult population in Delhi. These problems affect nearly half of the population. Overweight and obesity were more common in urban areas, while underweight was more often seen in rural areas. In the urban areas, overweight or obesity was prevalent in about 30% of the subjects compared to underweight observed in about 20% of the subjects. In the rural areas, underweight was the major nutritional problem affecting more than one-third of the population, while overweight and obesity were prevalent in only about 10% of the subjects. The prevalence of underweight and overweight or obesity was nearly similar in both sexes in rural areas, while females were more likely to be overweight or obese and less likely to be underweight in urban areas. The economic status had a significant impact on the nutritional status with the economically-better-off subjects being more likely to be overweight or obese and those with the lowest incomes were more likely to be underweight.

Some information on the nutritional profile is available from other parts of India. In the National Family Health Survey 2 (9), a mean BMI of 20.3 (rural–19.6 and urban–22.1) was reported in females in the reproductive age-group. The highest BMI was observed in Delhi (23.7) and the lowest in Orissa (19.2). In subjects aged over 35 years from urban Mumbai (16), a mean BMI of 21.8 in males and 22.7 in females was observed. Males in northeastern states had a mean BMI ranging from 18.3 to 20.5 (14). In urban women of five Indian cities in different states, the mean BMI ranged from 22.5 to 23.3 (13). From Kashmir in North India, a mean value of 22.3 in males and 23.88 in females has been reported (15). In the present study, the mean BMI was 22.75 in urban subjects (22.00 in males, 23.29 in females) and 20.16 in rural subjects (20.17 in males, 20.15 in females). A consistent observation in all studies, including the present one, was that females have a higher BMI than males. In agreement with the National Family Health Survey (1998) and the Kashmir study, urban subjects in the present study had a higher BMI compared to those from rural areas (9,15)

Data from other countries show that the mean BMI is higher in developed countries compared to developing countries (19). It ranges in males from 25.9 in the USA to 27.5 in Lithuania, and in females from 24.7 in the USA to 29.9 in Lithuania. Among developing countries, it is lowest in Tanzania (20.8 in men and 21.7 in women) and highest in Chile (21.7 in men and 21.2 in women).

The mean BMI does not adequately describe the distribution in the population. Therefore, the 5^th^, 50^th^, 85^th^, and 95^th^ percentiles in both sexes in different age intervals were also computed in the present study as suggested by the WHO Expert Committee (18). Similar information is not available in any other studies from India. The present data were compared with the U.S. National Health and Nutrition Examination Survey (NHANES) I that has been suggested as the international reference (20). It was observed that both males and females had lower values than whites and blacks for these percentiles in most age-groups. Further, the BMI peaked by 55 years of age in NHANES I data. Our data are in agreement with this observation.

Results of studies carried out in India showed that at least one-third or more of the rural population are undernourished: 33%–46% for males and 33%–47% for females. The prevalence of underweight has been found to be more variable in urban areas: 13%-37% for males and 11%-39% for females (9–11,14,16,21). The present study found an overall mean prevalence of underweight of 20.5% in urban areas (23% in males and 18.7% in females) and 38.5% in rural areas (38.1% in males and 38.7% in females). Thus, underweight is much more prevalent in rural areas. However, it appears to be equally common in males and females. Surprisingly, in the five-city study confined to urban women only (13), a much lower prevalence of underweight was observed with a mean of 5%. A different sampling strategy, confining to a better-off stratum of the community may be responsible for this observation.

Similarly, the prevalence of overweight or obesity showed considerable variations within the country: 7%–36.5% in urban males, 11%–50% in urban females, 3%-8% in rural males, and 7%-11% in rural females (9–11,12,14–16,21,22). In the present study, the overall mean prevalence of overweight/obesity was 25.5%. In urban areas, it was 30.2% (males–23.1%, females–35.3%) and 10.4% (males–10.9%, females–10.1%) in rural areas. In contrast to underweight, overweight and obesity are a much greater problem in urban areas. Although the females had a higher prevalence in urban areas, the prevalence was nearly similar in rural males and females.

There is some information on the risk factors associated with underweight and overweight or obesity. In the present study, we observed from multivariate analysis that older subjects, females, urban residents, and those from the higher-economic group had greater odds for being overweight or obese. In a similar analysis of the National Family Health Survey 2 data from the southern state of Andhra Pradesh, socioeconomic status was a more important predictor of both overweight and underweight than location of residence (6). Gopalan (12) and Reddy (23) also observed a greater prevalence of obesity in the higher-income groups. Interestingly, studies have repeatedly shown that high socioeconomic status is negatively associated with obesity in developed countries but positively correlated with it in developing countries. (24).

The WHO MONICA study has generated one of the most comprehensive datasets on the prevalence of obesity worldwide (25). About 15% of men and 22% of women are obese in European countries. The most recent data from the USA show that the prevalence of obesity is 10–20% in men and 10–25% in women (26). In general, developed countries have a higher prevalence of overweight and obesity. The secular trends suggest a definite increase (2). While the problem of obesity is of a lesser magnitude in developing countries, the increase observed over the years even in these regions is a cause for concern.

The study has limitations. The sample is not representative of the population of Delhi as the rural areas that constitute about 10% of the population are over-represented. However, as the selected areas were scattered across the city and each had a wide socioeconomic spectrum, it may reflect the true situation in Delhi as far as the distribution of BMI is concerned. The disproportionately larger rural sample provided an opportunity to compare urban-rural areas.

As shown in the present study, the problem of overweight and obesity is significant especially in urban areas and among females and co-exists with a high prevalence of underweight subjects. This agrees with the data of Berrios et al. from other developing countries showing the co-existence of both the nutritional problems (19). The burden of obesity shifts towards the groups with lower socioeconomic status as the gross national product of the country increases (27). India appears to be in a stage of nutritional transition, especially in urban areas. In the backdrop of early origin of adult disease, nutrition transition poses a major challenge for the future (28,29). Efforts at the national level are needed to address the problem of overnutrition on one hand and combat undernutrition on the other.
